# Correlation of serum albumin concentration with length of stay in Surgical Site Infection (SSI) patient at Rspad Gatot Soebroto, Jakarta, 2019-2022: a quantitative study

**DOI:** 10.1017/ash.2025.132

**Published:** 2025-09-03

**Authors:** Salsabila Luthfi Kusumaningati, Soroy Lardo, Retno Yulianti, Marlina Dewi Astuti, Martaviani Budiastuti

**Affiliations:** 1 Faculty of MedicineUniversitas Pembangunan Nasional “Veteran” Jakarta, Jakarta, Indonesia; 2 Division of Tropical and Infectious Diseases, Department of Internal MedicineGatot Soebroto Army Hospital, Jakarta; 3 Department of Pathology AnatomyUniversitas Pembangunan Nasional “Veteran” Jakarta, Jakarta, Indonesia; 4 Department of Internal MedicineUniversitas Pembangunan Nasional “Veteran” Jakarta, Jakarta, Indonesia; 5 Head of PPI CommitteeGatot Soebroto Army Hospital, Jakarta

**Keywords:** Surgical Site Infection (SSI), Albumin Concentration, Length of Stay, Indonesia

## Abstract

**Background:** The prevalence of surgery in Indonesia is increasing every year and may increase the prevalence of surgical site infection (SSI). SSI is an infection in the surgical site organ or space that occurs after surgery. Complex treatment of SSI has a significant impact on patient outcomes due to increased length of stay. There are a variety of risk factors, both endogenous and exogenous, that can affect the length of stay of SSI patients, especially the concentration of serum albumin before and after surgery. Albumin is an important component of proteins. Albumin plays a role in promoting inflammation, so tissue repair is done more quickly, and without albumin, the body is more difficult to carry out cell regeneration. This study aimed to determine the relationship between pre- and post-operative concentrations of albumin and duration of stay in SSI patients. **Method:** The study design used a quantitative study using cross-sectional secondary data from the medical records of 40 patients diagnosed with SSI at Gatot Soebroto Army Hospital. All SSI patients met the inclusion criteria. **Results:** The results showed that patients had moderate hypolbuminemia before surgery (35%) and after surgery (35%), long-term stay (50%), 19-60 years (77,5%), women (52,5%), comorbidities (50%), malnourished nutrition (60%), ASA score 2 (52,5%), clean surgical wound type (60%), abdominal or vaginal hysterectomy (17,5%), and showed that it has the characteristics of a normal operation period. (65%) Bivariate analysis using assay chi-squared shows a relationship between pre-operative serum albumin (p-value =0.005; PR=7.207; 95% CI=1.09-47.55) and post-operative (p-value=0.016; PR=3.857; 95% CI=1.05-14.08) with duration of stay in SSI patients. Concentration. Multivariate results indicate serum albumin preoperative concentration (p-value = 0.049). **Conclusion:** It can be concluded that serum albumin preoperative concentration is the only variable that greatly affects the length of stay of SSI patients.

